# The Patatin–Like Phospholipase Domain Containing Protein 7 Regulates Macrophage Classical Activation through SIRT1/NF-κB and p38 MAPK Pathways

**DOI:** 10.3390/ijms232314983

**Published:** 2022-11-29

**Authors:** Zheng Zhao, Christoph Heier, Huimin Pang, Yu Wang, Feifei Huang, Pingan Chang

**Affiliations:** 1 Chongqing Key Laboratory of Big Data for Bio-Intelligence, School of Bio-Information, Chongqing University of Posts and Telecommunications, Chongqing 400065, China; 2 Institute of Molecular Biosciences, University of Graz, 8010 Graz, Austria

**Keywords:** classical macrophage activation, PNPLA7, SIRT1, NF-κB, p38 MAPK

## Abstract

Lysophosphatidylcholine (LPC) is a bioactive lipid that modulates macrophage polarization during immune responses, inflammation, and tissue remodeling. Patatin-like phospholipase domain containing protein 7 (PNPLA7) is a lysophospholipase with a preference for LPC. However, the role of PNPLA7 in macrophage polarization as an LPC hydrolase has not been explored. In the current study, we found that PNPLA7 is highly expressed in naïve macrophages and downregulated upon lipopolysaccharide (LPS)-induced polarization towards the classically activated (M1) phenotype. Consistently, overexpression of PNPLA7 suppressed the expression of proinflammatory M1 marker genes, including interleukin 1β (IL-1β), IL-6, inducible nitric oxide synthase (iNOS), and tumor necrosis factor α (TNF-α), whereas knockdown of PNPLA7 augmented the inflammatory gene expression in LPS-challenged macrophages. PNPLA7 overexpression and knockdown increased and decreased Sirtuin1 (SIRT1) mRNA and protein levels, respectively, and affected the acetylation of the nuclear factor-kappa B (NF-κB) p65 subunit, a key transcription factor in M1 polarization. In addition, the levels of phosphorylated p38 mitogen-activated protein kinase (MAPK) were suppressed and enhanced by PNPLA7 overexpression and knockdown, respectively. Taken together, these findings suggest that PNPLA7 suppresses M1 polarization of LPS-challenged macrophages by modulating SIRT1/NF-κB- and p38 MAPK-dependent pathways.

## 1. Introduction

Macrophages are a heterogeneous population of innate myeloid cells with important roles in immunity and inflammation. Distinct stimuli promote the differentiation of macrophages into subsets with different phenotypes and functions [1]. Traditionally, two activation states of macrophages have been defined, namely, classically activated (M1) and alternatively activated (M2), which mirror the polarization of T cells toward T-helper type 1 (Th1) and type 2 (Th2) cells, respectively [2,3]. Specific cytokines initiate the differentiation of M1 or M2 macrophages. For example, granulocyte-macrophage colony-stimulating factor (GM-CSF) stimulates M1 differentiation and macrophage colony-stimulating factor (M-CSF) stimulates M2 differentiation [4]. Lipopolysaccharide (LPS), interferon-γ (IFN-γ), and tumor necrosis factor α (TNF-α) often prime initially differentiated macrophages toward the M1 phenotype, whereas M2 macrophages are induced by interleukin 4 (IL-4) or IL-13 [4,5]. M1 macrophages produce proinflammatory mediators, including IL-1β, IL-6, and TNF-α, and express high levels of inducible nitric oxide synthase (iNOS, NOS2), which has tumoricidal and microbicidal activity [4,5]. In contrast, M2 macrophages upregulate scavenger, mannose, and galactose receptors and the IL-1 receptor antagonist and downregulate IL-1β and other proinflammatory cytokines, exhibiting anti-inflammatory functions [2].

Lipids can act as signaling molecules to modulate inflammation and immune responses. Lysophosphatidylcholine (LPC) is an immunomodulatory lipid, which is abundant in the plasma where it is bound to albumin and lipoproteins [6,7]. Altered LPC plasma levels in patients with diabetes and atherosclerosis have been attributed a proinflammatory role in disease progression [6,7]. This notion is based on the observation that LPC affects immune cell functions including chemotaxis, cell adhesion, migration, and inflammatory activity [6,7]. LPC is also a major component of oxidized low-density lipoprotein (oxLDL) and has been detected at high levels in atherosclerotic lesions [6,7]. LPC exerts its action by modulating the activity of G-protein coupled receptors (directly or indirectly) and second messenger systems, although the exact mechanism of action remains incompletely understood [6].

Several in vitro studies illustrate a possible role of LPC in macrophage function and polarization [8,9,10,11]. Exogenous LPC stabilized M1 polarization and proinflammatory cytokine expression in human macrophages via the G protein coupled receptor (GPCR) G2A [9,10]. L-α-LPC promoted M1 activation of Th1-derived macrophages via calcium influx, which was suppressed by potent and selective inhibitors of transient receptor potential ankyrin 1 (TRPA1) [11]. Conversely, schistosomal-derived LPCs facilitated M2 polarization of murine macrophages through a peroxisome proliferator-activated receptor-γ (PPARγ)-dependent pathway [12]. Thus, how LPC affects macrophage polarization may depend on the cellular environment and distinct properties of LPC including concentration, acyl chain length, and saturation and whether it is bound to proteins or not.

LPC metabolism is coordinated by various enzymes that exist in the plasma or inside cells. Phospholipases A_1_ and A_2_ (PLA_2_) hydrolyze phosphatidylcholine (PC) to LPC [13]. Plasma LPC is also generated by lecithin-cholesterol acyltransferase (LCAT) [13]. LPC acyltransferases (LPCATs) consume LPC by converting it to PC using acylCoA as a donor [13]. In addition, LPC can be hydrolyzed by lysophospholipases A, C, or D to glycerophosphocholine (GPC), monoacylglycerol, and lysophosphatidic acid [13]. Several PLA and LPCAT isoforms have been implicated in macrophage polarization. Inhibition of the secreted enzyme lipoprotein-associated phospholipase A_2_ (Lp-PLA2) suppressed M1 polarization of human macrophages in vitro [14]. Depletion of LPCAT3, on the other hand, favored M1 differentiation [15,16,17]. These studies thus indicate that metabolism of LPC is an important determinant of macrophage polarization.

We previously identified patatin-like phospholipase domain containing protein 7 (PNPLA7) as an intracellular lysophospholipase A, which hydrolyzes LPC to GPC and free fatty acid (FFA) [18]. PNPLA7 shares high homology with the neuropathy target esterase (NTE)/PNPLA6, a phospholipase/lysophospholipase that converts PC and LPC to GPC [19,20]. PNPLA6 is highly expressed in the brain and PNPLA6 dysfunction has been linked to a spectrum of neurodegenerative syndromes [20]. Murine PNPLA7 is broadly expressed in peripheral tissues including testis, skeletal muscle, heart, and adipose tissue, and *Pnpla7* mRNA expression is regulated by insulin and nutritional status [21,22]. PNPLA7 is anchored in the endoplasmic reticulum (ER) membrane via its N-terminal transmembrane domain and interacts with cytosolic lipid droplets via its C-terminal catalytic domain [18,23]. PNPLA7 prefers LPC in vitro, and overexpression of PNPLA7 selectively reduces intracellular LPC levels in COS-7 cells [18,21]. Nevertheless, PNPLA7 appears to have functions beyond LPC hydrolysis, as it was recently shown to interact with apolipoprotein E (ApoE) and modulate ApoE stability and VLDL secretion independent of its catalytic activity [24].

Whether the LPC hydrolase PNPLA7 is expressed in macrophages and affects macrophage polarization is still unclear. Therefore, a potential role of PNPLA7 in macrophage polarization was explored. We found that PNPLA7 is highly expressed in naïve murine macrophages and downregulated during macrophage M1 polarization. Furthermore, we show that manipulation of PNPLA7 expression modulates classical activation and proinflammatory properties of macrophages by altering Sirtuin1 (SIRT1)/nuclear factor-kappa B (NF-κB) and p38 mitogen-activated protein kinase (MAPK)-dependent pathways. These results reveal a modulating role of PNPLA7 in M1 macrophage polarization.

## 2. Results

### 2.1. Pnpla7 Gene Expression Is Downregulated during Macrophage M1 Polarization

**Figure 1:**
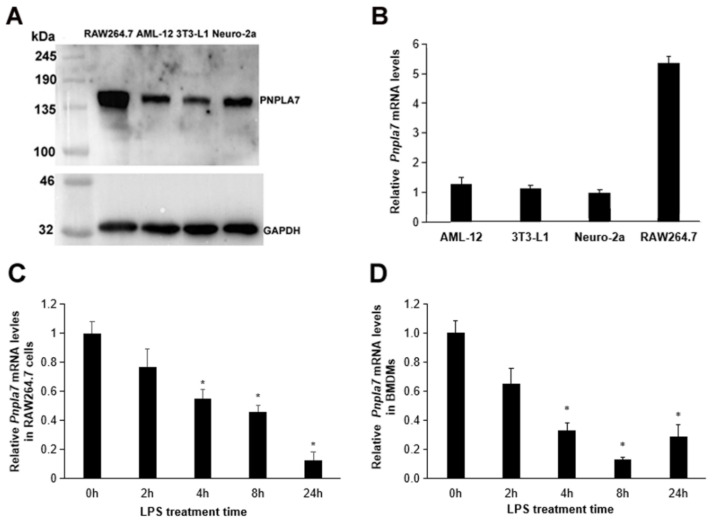
PNPLA7 expression in macrophages. (**A**) PNPLA7 protein expression in different murine cell lines was detected by immunoblotting analysis with an anti-PNPLA7 antibody. GAPDH was used as a loading control. (**B**) mRNA levels of the *Pnpla7* gene were detected by RT-qPCR in different murine cell lines. (**C**,**D**) mRNA expression of the Pnpla7 gene in RAW 264.7 cells and BMDMs after different times of LPS stimulation. Data are means ± SD generated from 5 independent measurements: *, significant difference from corresponding naïve (0 h) group; *p* < 0.05; *n* = 5.

First, we compared the expression of PNPLA7 in various mouse cells, including RAW264.7 macrophages, AML-12 hepatocytes, 3T3-L1 preadipocytes, and Neuro-2a neuroblastoma cells, by immunoblotting analysis. For the detection of endogenous PNPLA7 protein levels we used a rabbit antiserum directed towards a specific peptide within the regulatory domain of murine PNPLA7 [17]. As shown in Figure 1A, the protein levels of PNPLA7 in RAW264.7 cells were significantly higher as compared to other cells. Consistently, RT-qPCR also revealed higher *Pnpla7* mRNA levels in RAW264.7 compared to AML-12, 3T3-L1, and Neuro-2a cells (Figure 1B). Then, RAW264.7 macrophages were treated with 100 ng/mL LPS to induce polarization toward the M1 phenotype. Compared with naïve RAW264.7 macrophages, the mRNA expression of *Pnpla7* was gradually downregulated upon LPS challenge (Figure 1C). Likewise, LPS treatment of murine bone-marrow-derived macrophages (BMDMs) downregulated *Pnpla7* mRNA expression as compared to the naïve state (Figure 1D). These results indicate a role of PNPLA7 in M1 polarization of macrophages.

### 2.2. Overexpression of PNPLA7 Suppresses Proinflammatory Characteristics during M1 Polarization of Macrophages

**Figure 2:**
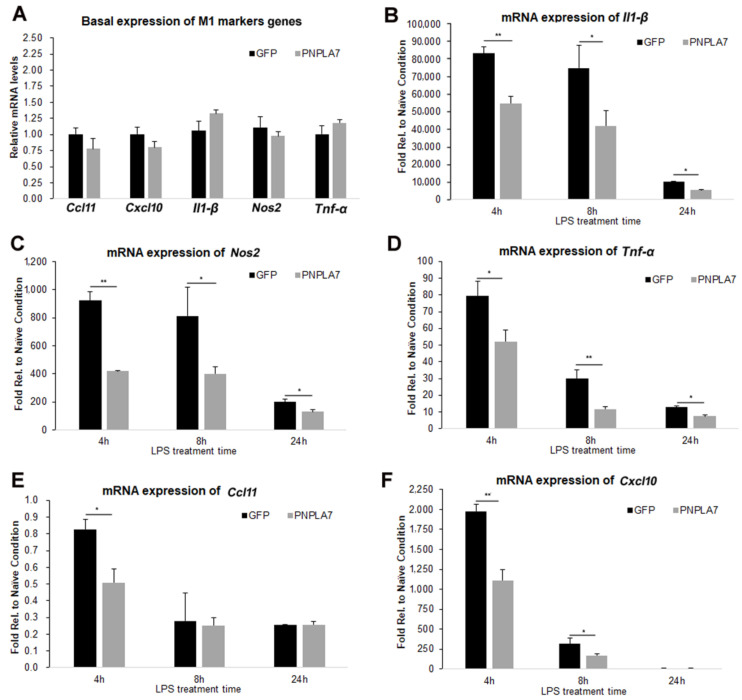
Impact of PNPLA7 overexpression on M1-related proinflammatory marker gene expression in RAW264.7 macrophages. Macrophages stably expressing GFP or PNPLA7-GFP (PNPLA7) were harvested under naïve conditions or at different timepoints after LPS stimulation and expression of proinflammatory M1 marker genes was assessed by RT-qPCR. (**A**) Relative mRNA expression of M1-related marker genes in naïve RAW264.7 expressing GFP or PNPLA7-GFP. (**B**–**F**) Induction of *Il1-β*, *Nos2*, *Tnf-α*, *Ccl11*, and *Cxcl10* mRNA expression by LPS treatment in GFP- or PNPLA7-GFP-expressing RAW264.7. Levels were expressed relative to naïve GFP-expressing cells. Data are means ± SD of *n* = 5 independent measurements: *, *p* < 0.05; **, *p* < 0.01.

We next examined whether overexpression of PNPLA7 affects M1 polarization of macrophages. To this end, we created RAW264.7 macrophages stably expressing PNPLA7-GFP or GFP alone. Stable expression of PNPLA7-GFP or GFP was confirmed by immunoblotting analysis using an anti-GFP antibody (Supplementary Figure S1). LPS treatment induces high levels of proinflammatory cytokines such as TNF-α and IL-1β, chemokines including C-C motif chemokine ligand 11 (CCL11) and C-X-C motif chemokine ligand 10 (CXCL10), and NOS2 that catalyzes the formation of nitric oxide [3]. To test if PNPLA7 modulates this response we stimulated RAW264.7 macrophages expressing PNPLA7-GFP or GFP alone with LPS and analyzed the expression of various M1-related marker genes. Under naïve conditions, mRNA levels of *Il-1β*, *Nos2*, *Tnf-α*, *Ccl11*, and *Cxcl10* were similar in PNPLA7-GFP-expressing and GFP-expressing macrophages (Figure 2A). However, when macrophages were stimulated with LPS, the induction of *Il-1β*, *Nos2*, and *Tnf-α* was significantly blunted in PNPLA7-GFP-expressing macrophages compared with GFP-expressing cells at 4 h, 8 h, and 24 h of the treatment (Figure 2B–D). Similarly, mRNA levels of *Ccl11* and *Cxcl10* were lower in PNPLA7-GFP-expressing RAW264.7 cells compared to GFP-expressing cells early after LPS treatment (Figure 2E,F). These data indicate that overexpression of PNPLA7 suppresses proinflammatory characteristics of macrophages polarized toward the M1 status.

### 2.3. PNPLA7 Stabilizes Sirt1 Expression to Restrict Acetylation of NF-κB/p65

**Figure 3:**
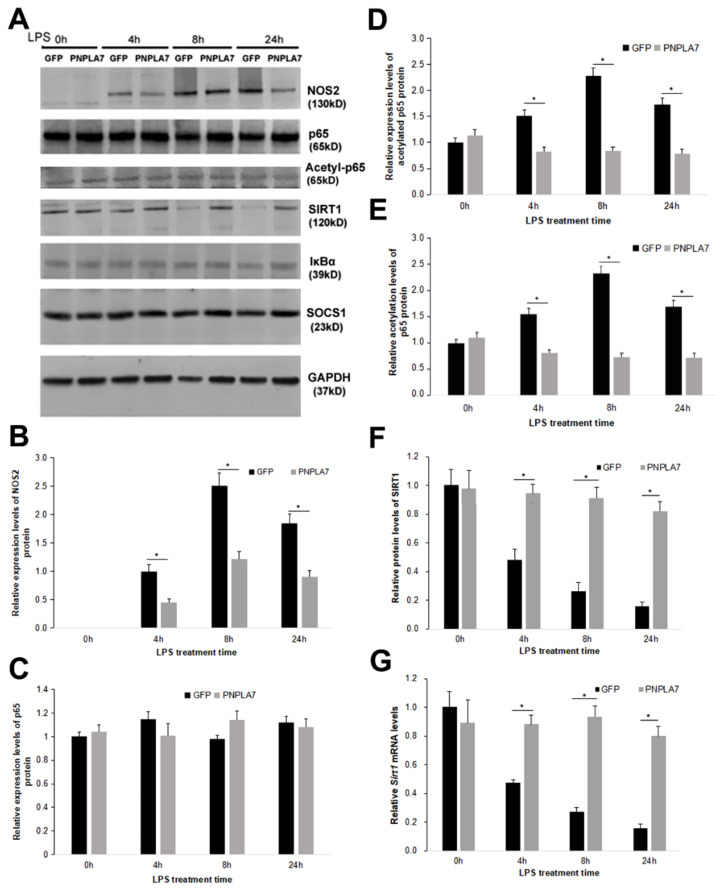
Impact of PNPLA7 overexpression on the expression of SIRT1 and the acetylation status of the NF-κB p65 subunit. (**A**) RAW264.7 cells expressing GFP or PNPLA7-GFP (PNPLA7) were harvested at different timepoints after LPS stimulation. The expressions of NOS2, SIRT1, acetylated p65 (acetyl-p65), p65, IκBα, SOCS1, and GAPDH were detected by immunoblotting analysis. (**B**–**F**) Quantification of NOS2, p65, acetylated p65, p65 acetylation, and SIRT1 protein levels. (**G**) mRNA expression of the *Sirt1* gene was detected by RT-qPCR analysis. Data are means ± SD: *, *p* < 0.05; *n* = 5.

How does PNPLA7 overexpression modulate LPS-induced macrophage polarization toward the M1 phenotype? LPS binding to Toll-like receptor 4 (TLR4) activates the transcription factor nuclear factor-kappa B (NF-κB) via a canonical signaling pathway. This pathway has a key role in LPS-induced macrophage polarization [25]. NF-κB signaling promotes transcription of proinflammatory cytokines, such as IL-1β, TNF-α, and IL-6, as well as NOS2 [26]. SIRT1 is an important modulator of this pathway, as it suppresses NF-κB-dependent transcription by deacetylation of the NF-κB p65 subunit [27]. We therefore investigated the acetylation status of NF-κB, protein levels of SIRT1, and the effector protein NOS2 by means of immunoblotting analysis (Figure 3A). NOS2 protein expression was undetectable in naïve macrophages but was induced during LPS-stimulated M1 polarization in GFP- and PNPLA7-GFP-expressing macrophages. However, the levels of NOS2 were significantly reduced in PNPLA7-GFP-expressing macrophages at the indicated times of LPS stimulation compared with GFP-expressing macrophages (Figure 3A,B). The protein levels of NF-κB p65 were not significantly different between PNPLA7-GFP- and GFP-expressing macrophages (Figure 3A,C). However, LPS stimulation was associated with an increase in acetylated p65 subunit in GFP-expressing control macrophages but not in PNPLA7-GFP-expressing macrophages (Figure 3A,D). As a consequence, p65 acetylation levels (acetylated-p65/total p65) were reduced in PNPLA7-GFP-expressing macrophages as compared to controls at 4 h, 8 h, and 24 h after LPS stimulation (Figure 3E). Moreover, protein expression of the p65 deacetylase SIRT1 decreased in LPS-challenged control macrophages but not in PNPLA7-GFP-expressing macrophages (Figure 3A,F). This resulted in increased SIRT1 protein expression in PNPLA7-GFP-expressing macrophages throughout LPS stimulation (Figure 3A,F). Consistently, mRNA expression of *Sirt1* was also significantly higher in PNPLA7-GFP-expressing macrophages as compared to controls at 4 h, 8 h, and 24 h of LPS treatment (Figure 3G). We finally assessed the protein expression of an inhibitor of NF-κB (IκB) and a suppressor of cytokine signaling-1 (SOCS1), which have been shown to suppress NF-κB activity and proinflammatory characteristics of M1 macrophages [26]. However, as shown in Figure 3A, protein expressions of IκB and SOCS1 were similar in PNPLA7-GFP-expressing and control macrophages throughout the indicated times of LPS treatment. These results indicate that PNPLA7 suppresses proinflammatory characteristics of LPS-challenged macrophages by stabilizing the expression of SIRT1 and inhibiting p65 acetylation.

### 2.4. Knockdown of Pnpla7 Favors Proinflammatory Characteristics during M1 Polarization of Macrophages

**Figure 4:**
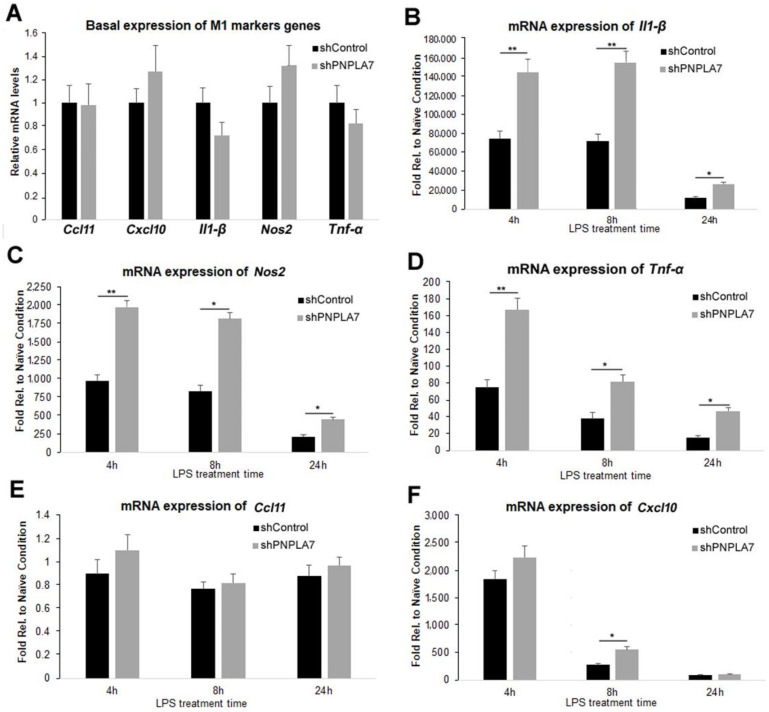
Impact of PNPLA7 knockdown on M1-related marker gene expression in RAW264.7 macrophages. RAW264.7 macrophages expressing shRNA targeting PNPLA7 (shPNPLA7) or control shRNA (shControl) were harvested under naïve conditions and at the indicated timepoints after LPS treatment and mRNA expression of M1-related marker genes was analyzed by RT-qPCR. (**A**) Relative mRNA levels of M1 marker genes in naïve RAW264.7 macrophages. (**B**–**F**) Induction of *Il1-β*, *Nos2*, *Tnf-α*, *Ccl11*, and *Cxcl10* mRNA expression during LPS treatment in shPNPLA7- and shControl-expressing RAW264.7 macrophages. Levels were expressed relative to naïve shControl-expressing cells. Data are the means ± SD of *n* = 5 independent measurements: *, *p* < 0.05; **, *p* < 0.01.

To examine whether inhibition of PNPLA7 expression influences macrophage M1 polarization, we silenced endogenous *Pnpla7* expression in RAW264.7 macrophages by shRNA and assessed the expression of M1-related marker genes after LPS challenge. Two independent shRNA constructs targeting *Pnpla7* (shPNPLA7-1 and shPNPLA7-2) reduced *Pnpla7* mRNA levels by 78% and 50%, respectively, compared with cells expressing control shRNA (shControl) (Supplementary Figure S2A). Conversely, the expression of the closely related *Pnpla*6 gene was not affected by shPNPLA7-1 or shPNPLA7-2 (Supplementary Figure S2B). Thereafter, macrophages stably expressing shPNPLA7-1, named shPNPLA7, were selected for the following experiments. Macrophages were stimulated with LPS for different durations and expression of various M1-related marker genes was analyzed by RT-qPCR. Under naïve conditions, mRNA levels of *Il-1β*, *Nos2*, *Tnf-α*, *Ccl11*, and *Cxcl10* were not altered in shPNPLA7- compared with shControl-expressing macrophages (Figure 4A). LPS treatment substantially increased mRNA expression of M1 marker genes as compared with naïve conditions, but the inductions of *Il-1β*, *Nos2*, and *Tnf-α* were significantly higher in shPNPLA7-expressing macrophages compared to shControl-expressing macrophages at 4 h, 8 h, and 24 h of LPS treatment (Figure 4B–D). Knockdown of *Pnpla7* did not affect mRNA expression of *Ccl11* and only moderately increased mRNA levels of *Cxcl10* compared to shControl-expressing cells at 8 h after LPS treatment (Figure 4E,F). These findings suggest that silencing of *Pnpla7* expression promotes proinflammatory characteristics of the M1 phenotype in LPS-challenged macrophages.

### 2.5. Reduction of PNPLA7 Downregulates SIRT1 to Promote Acetylation of NF-κB/p65

**Figure 5:**
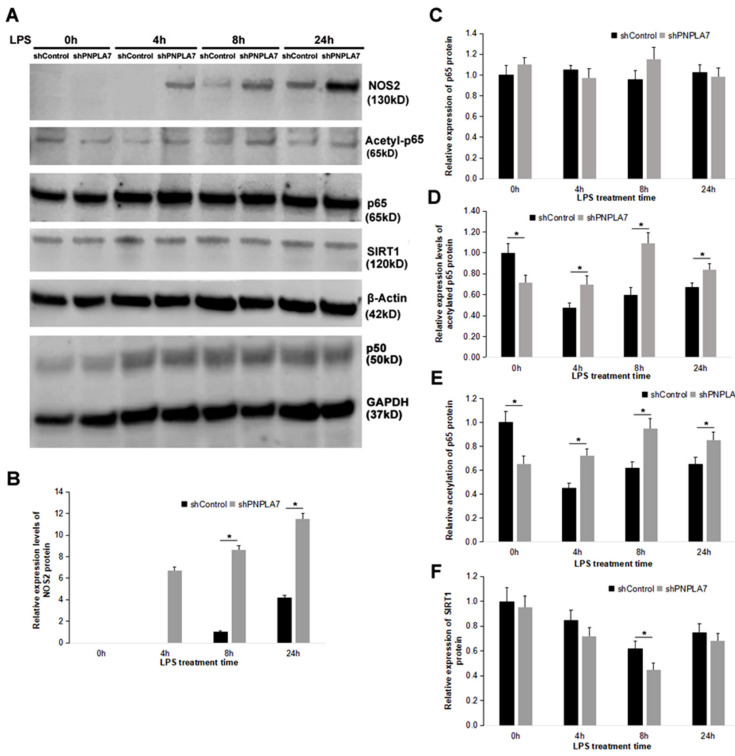
Impact of PNPLA7 knockdown on SIRT1 protein expression and the acetylation status of the NF-κB p65 subunit. (**A**) The protein expression of NOS2, acetyl-p65, p65, SIRT1, β-actin, p50, and GAPDH was detected by immunoblotting analysis. (**B**–**F**) Quantification of NOS2, p65, acetylated p65, acetyl-p65/p65, and SIRT1 protein levels. Data are the means ± SD: *, *p* < 0.05; *n* = 5.

**Figure 6:**
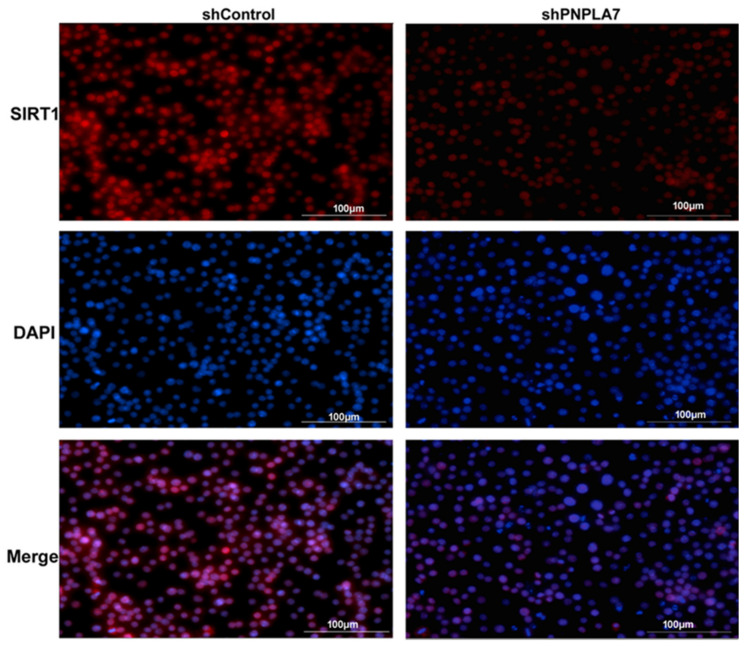
Immunofluorescence analysis of SIRT1 expression and localization in RAW264.7 macrophages. Cells treated with LPS for 8 h were fixed with formaldehyde and permeabilized with ice-cold 100% methanol and then blocked. The expression of SIRT1 (red) was analyzed by immunofluorescence through incubation with anti-SIRT1 antibody and a secondary antibody coupled to Alexa Fluor^®^ 647. Nuclei were stained with DAPI (blue). Fluorescent images were obtained with BioTek’s Lionheart LX Automated Microscope. All the presented experiments were repeated independently at least three times. Scale bar = 100 μm.

We next asked whether the SIRT1/NF-κB pathway is involved in modulation of macrophage M1 polarization upon silencing of *Pnpla7*. As shown in Figure 5A,B, NOS2 protein levels were significantly increased in shPNPLA7-expressing macrophages at 4 h, 8 h, and 24 h after LPS stimulation as compared to shControl-expressing macrophages, mimicking increased *Nos2* mRNA levels in these cells (Figure 4C). The expression levels of the total NF-κB p65 subunit were not influenced by the reduction of PNPLA7 (Figure 5C). In contrast, levels of the acetylated p65 subunit were decreased in naïve shPNPLA7-expressing macrophages as compared to controls but were increased at 4 h, 8 h, and 24 h after LPS treatment (Figure 5A,D). Therefore, knockdown of PNPLA7 decreased the acetylation of NF-κB p65 in the process of M1 macrophage polarization (Figure 5E). SIRT1 protein expression decreased upon LPS treatment in both shPNPLA7- and shControl-expressing macrophages (Figure 5A,F). Nevertheless, shPNPLA7-expressing macrophages exhibited significantly lower SIRT1 protein levels as compared to shControl-expressing cells at 8 h of LPS treatment (Figure 5E). Immunofluorescence analysis revealed that SIRT1 predominantly localized to the nucleus of both shPNPLA7- and shControl-expressing macrophages (Figure 6). Yet, total cellular SIRT1 immunofluorescence was markedly weaker in shPNPLA7- as compared to shControl-expressing macrophages (Figure 6). Protein levels of p50, another subunit of NF-κB, were upregulated to a similar extent in shPNPLA7- and shControl-expressing macrophages upon LPS stimulation (Figure 5A). We conclude that silencing of *Pnpla7* expression decreases SIRT1 protein expression, resulting in elevated NF-κB p65 subunit acetylation and increased proinflammatory characteristics during M1 polarization.

### 2.6. PNPLA7 Influences the Phosphorylation of p38 MAPK

**Figure 7:**
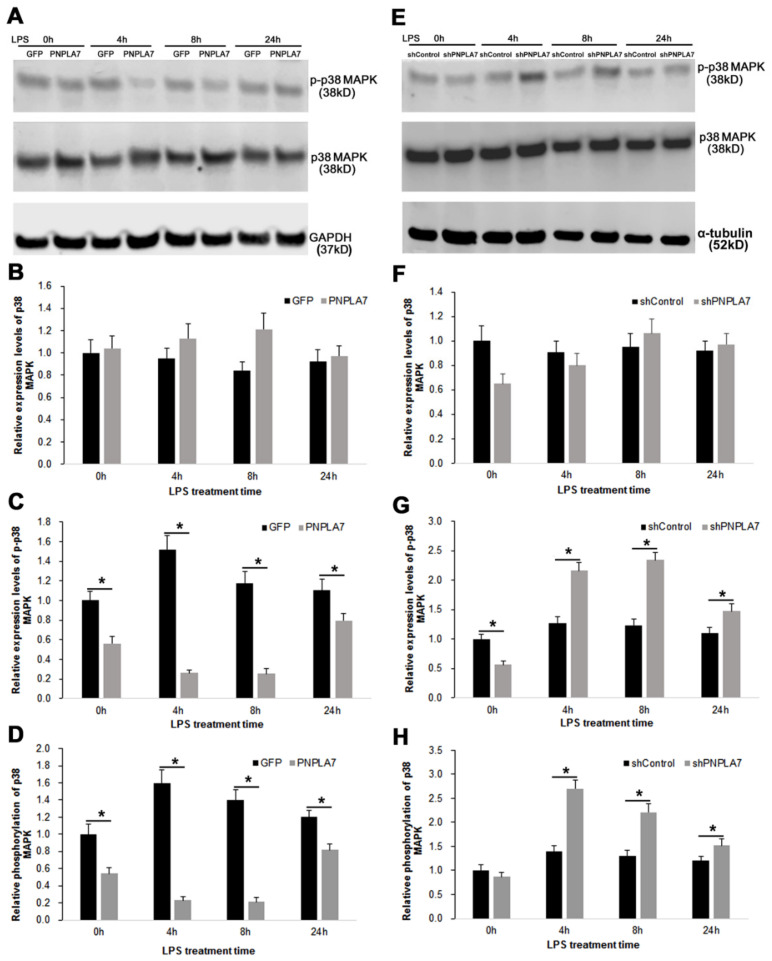
Overexpression and knockdown of PNPLA7 affects the phosphorylation of p38 MAPK in RAW264.7 macrophages: (**A**) and (**E**), the expression of phosphorylated-p38 MAPK (p-p38 MAPK), p38 MAPK, GAPDH, and α-tubulin was detected by immunoblotting analysis in (**A**) PNPLA7- vs. GFP-expressing RAW264.7 and (**E**) shPNPLA7- vs. shControl-expressing RAW264.7 macrophages; (**B**–**D**) and (**F**–**H**), quantification of p38 MAPK, p-p38 MAPK, and phosphorylation of p38 MAPK (p-p38 MAPK/p38 MAPK) levels. Data are the means ± SD: *, *p* < 0.05; *n* = 5.

Activation of the p38 mitogen-activated protein kinase (MAPK) is required for transcriptional activity of NF-κB and production of proinflammatory cytokines in macrophages. LPS/TLR4 signaling induces phosphorylation and activation of p38 MAPK in macrophages [28]. Once phosphorylated, p38 MAPK (p-p38 MAPK) regulates expression of a variety of proinflammatory cytokines and NOS2 [28]. We therefore asked whether altered p38 MAPK pathway activity is involved in PNPLA7-mediated modulation of inflammatory characteristics in LPS-challenged macrophages (Figure 7). Levels of total p38 MAPK were not significantly altered in PNPLA7-overexpressing cells compared to control macrophages in both the naïve state and upon LPS stimulation (Figure 7B,F). In contrast, the expression of p-p38 MAPK was downregulated and upregulated by overexpression and knockdown of PNPLA7, respectively, in the process of M1 macrophage polarization (Figure 7C,G). The phosphorylation of p38 MAPK (ratio of p-p38 MAPK to total p38 MAPK) was moderately increased in control macrophages as compared to the naïve state. The phosphorylation of p38 MAPK was significantly reduced in naïve PNPLA7-GFP-expressing macrophages as compared to GFP-expressing controls, whereas silencing of *Pnpla7* expression did not affect basal p38 MAPK phosphorylation (Figure 7D,H). However, after 4 h, 8 h, and 24 h of LPS stimulation, p38 MAPK phosphorylation levels were significantly increased and decreased in PNPLA7-GFP- and shPNPLA7-expressing cells, respectively, as compared to controls (Figure 7D,H). Thus, PNPLA7 modulates the polarization of macrophages towards M1 by affecting the phosphorylation of p38 MAPK.

## 3. Discussion

LPC is increasingly recognized as a potent immunomodulatory signaling lipid and has been shown to affect chemotaxis, phagocytosis, and inflammatory properties of macrophages [6,7,13]. However, the signaling pathways and downstream events involved in LPC bioactivity remain largely uncharacterized. Moreover, the metabolic pathways that control macrophage LPC levels remain poorly understood.

Our data identify the LPC hydrolase PNPLA7 as the modulator of macrophage M1 polarization. PNPLA7 belongs to the PNPLA family of proteins, which includes enzymes with lipase, transacylase, and (lyso)phospholipase activities [20,21]. The triacylglycerol lipase PNPLA2/ATGL and the phospholipase PNPLA9/iPLA2β are expressed in macrophages and influence key effector functions and inflammatory characteristics of this cell type [29,30]. In this study, we demonstrate that PNPLA7 likewise is expressed in macrophages and influences macrophage inflammatory properties. *Pnpla7* expression gradually decreased in LPS-treated BMDMs or RAW264.7 cells concomitantly with the manifestation of proinflammatory characteristics. Consistently, silencing of *Pnpla7* gene expression enhanced the proinflammatory signature of LPS-stimulated RAW264.7 cells while ectopic expression of PNPLA7-GFP had the opposite effect. This observation supports the conclusion that PNPLA7 acts as a suppressor of proinflammatory characteristics during LPS-induced M1 polarization. PNPLA7 prefers LPC in vitro and decreases cellular LPC content when expressed in cultured cells. Although it remains to be shown whether PNPLA7 regulates macrophage intracellular LPC levels or composition, these data support the notion that enzymatic control of intracellular LPC metabolism is a critical modulator of macrophage inflammatory properties during M1 polarization.

Macrophages express several additional enzymes with putative or documented roles in LPC metabolism including several PLA2, LPCAT, and lysophospholipase isoenzymes [16,31]. Specific PLA2 and LPCAT isoenzymes have been shown to regulate macrophage inflammatory characteristics during differentiation toward M1- or M2-related states. For example, Lp-PLA2 and LPCAT2 promote M1-related characteristics in different in vitro models of macrophage differentiation [14,32]. Conversely, LPCAT3 and group IVC phospholipase A2 have been shown to suppress M1 polarization [15,16,17,33]. LPCAT3 is a key component of the Land’s cycle in macrophages, as it preferentially esterifies LPC with polyunsaturated FAs including arachidonic acid [16]. Loss of LPCAT3 reduces polyunsaturated phospholipids and promotes LPS-induced cytokine production in murine BMDMs [17]. Thus, similar to PNPLA7, LPCAT3 suppresses macrophage proinflammatory characteristics during M1 polarization. It has been speculated that LPCAT3 affects TLR4 signaling by altering plasma membrane composition and biophysical properties rather than through modulation of bioactive LPC [17]. Thus, in the future it will be interesting to learn whether these enzymes act through a common mechanism (i.e., by consumption of bioactive LPC or alteration of membrane properties) or through distinct lipid classes.

The expression of proinflammatory genes in naïve RAW264.7 macrophages was largely unaffected by manipulations of *Pnpla7* expression. Thus, PNPLA7 appears not to regulate basal characteristics of macrophages but specifically modulates outcomes of TLR signaling. LPS binding to TLR4 triggers several signaling cascades including canonical NF-κB signaling, which results in NF-κB nuclear translocation and transcription of proinflammatory factors including the cytokines IL-1β, IL-6, and TNF-α [34]. The activity of this pathway is regulated by post-transcriptional modifications of NF-κB subunits including phosphorylation, acetylation, ubiquitination, and prolyl isomerization [35]. SIRT1 is able to deacetylate lysine 310 of the p65 subunit, thereby affecting its transcriptional activity and decreasing expression of its proinflammatory target genes [35]. SIRT1 mRNA and protein levels are downregulated in macrophages upon LPS-stimulation, and SIRT1 knockdown leads to an increase in inflammatory gene expression [36,37,38]. Silencing of *Pnpla7* expression in RAW264.7 macrophages accentuated LPS-induced downregulation of SIRT1 protein levels. Conversely, ectopic expression of PNPLA7-GFP robustly increased SIRT1 protein expression throughout LPS treatment and blunted acetylation of the NF-κB p65 subunit. This suggests that PNPLA7 modulates TLR4/NF-κB activity by affecting post-translational inputs via SIRT1. How does PNPLA7 regulate SIRT1 expression and/or activity? Thus far, relatively little is known about the regulation of SIRT1 by lipids. Omega-3 polyunsaturated FAs have been demonstrated to activate SIRT1 and suppress the inflammatory responses in LPS-stimulated macrophages and microglia [39,40,41]. Moreover, monounsaturated FAs have been shown to allosterically activate SIRT1 towards specific substrates such as peroxisome proliferator-activated receptor-γ coactivator 1-α [42]. PNPLA7 prefers unsaturated compared to saturated LPC species and may thus contribute to the formation of FAs regulating SIRT1 [18]. Thus, a detailed mapping of PNPLA7-dependent lipid metabolic pathways may provide important insights into the regulation of SIRT1 by lipids.

LPS/TLR4 signaling activates MAPK pathways, which play essential roles in the inflammatory response of macrophages [43]. The p38 MAPK pathway regulates proinflammatory cytokine expression and exhibits crosstalk with canonical NF-κB signaling [43]. In our LPS-challenged RAW264.7 model, PNPLA7 overexpression reduced whereas PNPLA7 knockdown increased the level of phosphorylated p38 MAPK. This indicates that, in addition to SIRT1, PNPLA7 modulates the MAPK branch of TLR4 signaling. It has been shown that exogenous LPC can promote the phosphorylation of p38 MAPK in macrophages even in the absence of LPS stimulation [44,45]. Our data indicate that modulation of intracellular LPC or LPC-related lipids by PNPLA7 likewise plays a critical role in modulating p38 MAPK activity.

## 4. Material and Methods

### 4.1. Materials

WT C57BL/6 mice aged from 6 to 8 weeks were obtained from the Experimental Animal Center of Chongqing Medical University. The lentiviral pLVX IRES Puro constructs to express GFP and PNPLA7-GFP Mission^®^ lentiviral pLKO.1 vectors encoding for scrambled shRNA or shRNAs targeting murine PNPLA7 were maintained in our lab [18]. LPS from *Escherichia coli* O111:B4 was obtained from Sigma. The MiniBEST Universal RNA Extraction Kit, PrimeScript™ II 1st Strand cDNA Synthesis Kit, and TB Green^®^ Fast qPCR Mix were purchased from TaKaRa. A PNPLA7 rabbit antiserum was generated in our lab by immunizing rabbits with a peptide in the regulatory domain of mouse PNPLA7. The generation of this antiserum and its specificity have been previously described [17]. The SIRT1 mouse monoclonal antibody (mAb) was purchased from Santa Cruz Biotechnology and β-actin mouse mAb, α-Tubulin mouse mAb, GAPDH mouse mAb, NF-κB p65 rabbit mAb, NF-κB1 p105/p50 rabbit mAb, iNOS antibody (Mouse Specific), IκBα (44D4) Rabbit mAb, p38 MAPK rabbit mAb, and Phospho-p38 MAPK (Thr180/Tyr182) rabbit mAb were obtained from Cell Signaling Technology. Anti-SOCS1 antibody and Anti-NF-κB p65 (acetyl K310) antibody were obtained from Abcam. Goat anti-Rabbit IgG (H + L) Highly Cross-Adsorbed Secondary Antibody Alexa Fluor^®^ 647 conjugate was purchased from Thermo Fisher Scientific.

### 4.2. Cell Culture and Treatment

Neuro-2a cells (ATCC CCL-131) were maintained in minimum essential medium (MEM) supplemented with 2 mM L-glutamine, 1 mM sodium pyruvate, non-essential amino acids, 10% fetal bovine serum (FBS), 100 units/mL penicillin, and 100 µg/mL streptomycin. RAW264.7 cells (ATCC CRL-1651) and 3T3-L1 (ATCC CL-173) cells were cultured in Dulbecco’s modified Eagle’s medium (DMEM) supplemented with 10% FBS, 100 units/mL penicillin, and 100 µg/mL streptomycin. AML12 cells (ATCC CRL-2254) were cultured in a 1:1 mixture of DMEM and Ham’s F12 medium supplemented with 10% FBS, 100 units/mL penicillin, 100 µg/mL streptomycin, 0.005 mg/mL insulin, 0.005 mg/mL transferrin, 5 ng/mL selenium, and 40 ng/mL dexamethasone. All cells were maintained at 37 °C, 95% humidity, and 5% CO_2_. RAW264.7 cells were stimulated with 100 ng/mL LPS for the indicated time toward an M1 status.

### 4.3. BMDM Culture and Treatment

Mice were kept on a 12 h/12 h light/dark cycle with standard chow. Mice were sacrificed by ether anesthesia and bone marrow was collected immediately. Macrophages derived from the bone marrow were cultured according to an established protocol [46]. Briefly, bone marrow was flushed from femur and tibia with culture media under aseptic conditions. Single-cell suspensions of bone marrow were then cultured in DMEM containing 10% heat-inactivated FBS, 100 U/mL penicillin, 100 µg/mL streptomycin, and 20% L929 cell-conditioned complete medium to prepare macrophages. Macrophages were collected using an 18-gage needle to spray cells with PBS containing 1 mM EDTA. Macrophages were plated for 24–48 h and stimulated with 100 ng/mL LPS for the indicated durations toward M1 status. Cells were treated with vehicle alone as native macrophages.

### 4.4. Generation of Lentivirus and Stable Infection of RAW264.7 Cells

According to the manufacturer’s instructions (Clontech, Mountain View, CA, USA), lentiviral particles harboring pLVX IRES Puro vectors for the expression of GFP and PNPLA7-GFP proteins or pLKO.1 vectors for the expression of shRNAs were generated in HEK293T cells. Before transduction, RAW 264.7 cells were seeded into 6-well plates at a density of 300,000 cells per well. Cells were incubated for 24 h with lentivirus-containing supernatants in the presence of 8 µg/mL polybrene. To select for stable expression, cells were maintained for 7 days in medium containing 2 µg/mL puromycin. The selected stable cell clones were verified by Western blotting or real-time quantitative PCR and maintained in DMDM culture containing 0.5 µg/mL puromycin.

### 4.5. Real-Time Quantitative PCR

Total RNA was extracted and genomic DNA was digested with DNase I during the process. Total RNA was then reverse transcribed to cDNA with random primers. Synthesized cDNA was diluted at a ratio of 1:10 as template. Real-time quantitative PCR (RT-qPCR) was performed with Fast SYBR^®^ Green Master Mix on a QuantStudio 3 Real-Time PCR System. The used primers are shown in Table 1 [17,46]. To account for differences in cell numbers, all cycle threshold (Ct) values of sample replicates were normalized to that of *36B4*. Relative mRNA levels are expressed as the fold change relative to controls with the ΔΔCt method [47].

### 4.6. Immunoblotting Analysis

Cells were harvested, briefly sonicated, and then lysed using RIPA buffer with protease and phosphatase inhibitors, followed by centrifugation at 15,000× *g* for 15 min at 4 °C. Total protein of the supernatant was determined by protein concentration assay with a Pierce BCA Protein Assay Kit, followed by immunoblotting. Samples were subjected to SDS-PAGE and electroblotted onto nitrocellulose membranes. Membranes were incubated with blocking buffer (TBST containing 5% BSA) for 1 h, followed by incubation with primary antibodies at 4 °C overnight. Antibodies were diluted in blocking buffer at a ratio of 1:1000, with the exception of anti-α-tubulin (1:5000), anti-β-actin (1:5000), and anti-PNPLA7 (1:10,000). After incubation with secondary HRP-conjugated antibodies, protein signals were detected by enhanced chemiluminescence. The blots were quantified by ImageJ (1.43) software.

### 4.7. Immunofluorescence Analysis

Cells seeded in 12-well plates were fixed with 4% formaldehyde in PBS for 15 min at room temperature and rinsed three times in PBS for 5 min each. Then, cells were permeabilized with ice-cold 100% methanol for 10 min at –20 °C and blocked in blocking buffer (1 × PBS/5% normal goat serum 0.3% Triton X-100) for 60 min. The cells were incubated with anti-SIRT1 antibody (1:100 dilution in 1 × PBS/1% BSA/0.3% Triton X-100 buffer) overnight at 4°C. After washing three times in PBS for 5 min each, the cells were incubated with goat anti-Rabbit IgG (H + L) Highly Cross-Adsorbed Secondary Antibody Alexa Fluor^®^ 647 conjugate at a concentration of 2 µg/mL in PBS containing 0.2% BSA for 1–2 h at room temperature in the dark. Then, 0.5 µg/mL DAPI was used for nuclear counterstaining. Immunofluorescence images were captured on a Lionheart LX automated microscope and analyzed using the Gen5 Microplate Reader and Imager Software (BioTek Instruments, Inc. Agilent, Santa Clara, CA, USA). DAPI was detected using a 405 nm LED and a DAPI filter cube and Alexa Fluor^®^ 647 was detected using a 623 nm LED and a CY5 filter cube. All the presented experiments were repeated independently at least three times.

### 4.8. Statistical Analysis

Data are presented as mean ± standard deviation (SD). Groups were compared by one-way ANOVA and by post hoc analysis using the Student–Keuls method. A significant difference between means was determined as *p* < 0.05.

## 5. Conclusions

**Figure 8:**
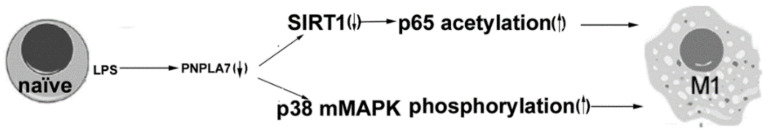
A proposed role of PNPLA7 in M1 macrophage polarization. Naïve macrophages are polarized toward an M1 phenotype by LPS, which suppresses the expression of PNPLA7. Reduction of PNPLA7 lowers SIRT1 levels, leading to the upregulation of NF-κB p65 subunit acetylation, and elevates the phosphorylation of p38 MAPK to facilitate M1 macrophage polarization.

In summary, our study for the first time illustrates a role of the lysophospholipase PNPLA7 in macrophage M1 polarization (Figure 8). Our data imply that PNPLA7 dampens proinflammatory gene expression downstream of LPS/TLR4 by modulating SIRT1 and p38 MAPK activity. Downregulation of *Pnpla7* expression thus appears to accentuate proinflammatory gene expression in LPS-challenged macrophages. Our findings add to the increasing number of studies suggesting an important immunomodulatory role of enzymes regulating LPC metabolism in macrophage biology.

## Figures and Tables

**Table 1:** Primers used to quantify mRNA levels.

Gene Name	Primer Sequence (5′–3′)
*Ccl11* *Cxcl10* *Il1β* *Nos2* *Pnpla7* *Pnpla6* *Sirt1* *Tnfα* *36B4*	GAATCACCAACAACAGATGCAC ATCCTGGACCCACTTCTTCTT GCCGTCATTTTCTGCCTCAT GCTTCCCTATGGCCCTCATT CCATGGCACATTCTGTTCAAA GCCCATCAGAGGCAAGGA CAGCTGGGCTGTACAAACCTT CATTGGAAGTGAAGCGTTTCG CGTGTT TTCCAACGACCACC TCTGCTAGTGCCCTGAGGAT CGGGTGCAGAAAACTCCAG CGCATAATCTTCCGGCCATAGA GTCACACGCCAGCTCTAGT GACAGAAACCCCAGCTCCA TTCGGCTACCCCAAGTTCAT CGCACGTAGTTCCGCTTTC GCTTCATTGTGGGAGCAGACA CATGGTGTTCTTGCCCATCAG

## Data Availability

Not applicable.

## Supplementary Materials

The following supporting information can be downloaded at: https://www.mdpi.com/article/10.3390/ijms232314983/s1.
