# Supplementary material for: The Patatin–Like Phospholipase Domain Containing Protein 7 Regulates Macrophage Classical Activation through SIRT1/NF-κB and p38 MAPK Pathways
Source: Int J Mol Sci. 2022 Nov 29;23(23):14983. doi: 10.3390/ijms232314983 (PMC9739533; doi:10.3390/ijms232314983)
Supplement: Supplementary file 1 [file ijms-23-14983-s001.zip › ijms-2030433-supplementary.pdf]

Supplementary Figures

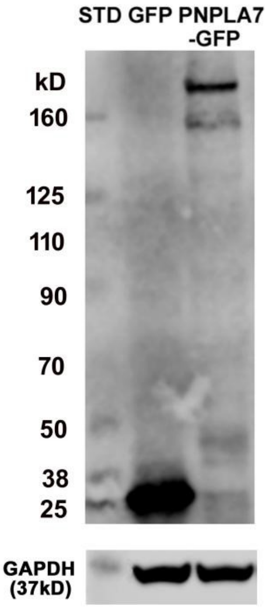

Figure. S1 Western blotting of GFP and PNPLA7-GFP in RAW264.7 cells using anti-GFP antibody. Migration of molecular mass standard proteins (STD) was indicated on the left of the figure. Figures are representative of three separate experiments.

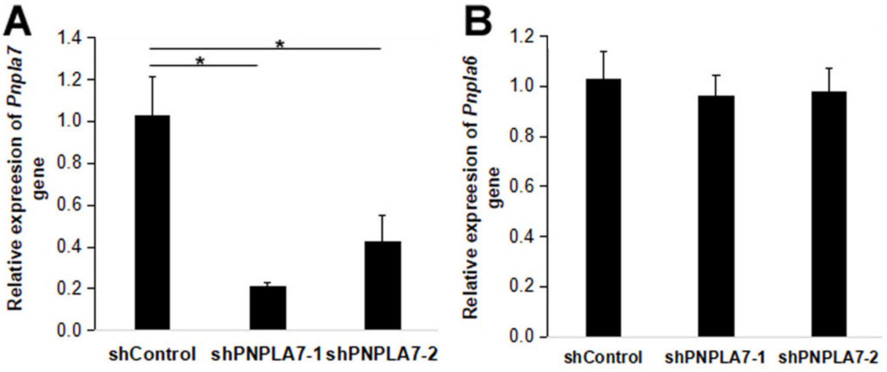

Figure S2 Relative mRNA expression of *Pnpla7* and *Pnpla6* genes in control (shControl) and PNPLA7 knockdown (shPNPLA7) RAW264.7 macrophages. Data was presented as the fold change of control cells. Data are the means ± SD. *, P <0.05, n = 5.
